# Impacts of *Nicotiana glauca* Graham Invasion on the Vegetation Composition and Soil: A Case Study of Taif, Western Saudi Arabia

**DOI:** 10.3390/plants10122587

**Published:** 2021-11-25

**Authors:** Abdulaziz M. Assaeed, Abdullah S. Alharthi, Ahmed M. Abd-ElGawad

**Affiliations:** 1 Plant Production Department, College of Food & Agriculture Sciences, King Saud University, P.O. Box 2460, Riyadh 11451, Saudi Arabia; assaeed@ksu.edu.sa; 2 Department of Botany, Faculty of Science, Mansoura University, Mansoura 35516, Egypt

**Keywords:** tree tobacco, plant invasion, vegetation dynamics, facilitation, competition

## Abstract

Invasive species are considered a serious problem in different ecosystems worldwide. They can compete and interfere with native plants, leading to a shift in community assembly and ecosystem function. The present study aimed to evaluate the effects of *Nicotiana glauca* Graham invasion on native vegetation composition and soil of the most invaded locations in the Taif region, Western Saudi Arabia, including Alwaht (WHT), Ar-Ruddaf (RDF), and Ash-shafa (SHFA). Plant species list, life span, life form, and chorotypes were assessed. Six locations highly infested with *N. glauca* shrubs were selected, and the morphological parameters of the shrubs were measured. Within each location, richness, evenness, relative density of species, and soil were measured either under the canopy of *N. glauca* shrubs or outside the canopy. Floristic analysis revealed the existence of 144 plant species, mainly perennial. The shrubs at the SHFA1 location showed the highest values of all measured morphological parameters. The WHT 1 location showed high richness and evenness, while the WHAT 2 location showed less richness and evenness. The invaded locations showed substantial variation in the community composition. Additionally, the effect of *N. glauca* on the understory species varied from competition to facilitation, where most of the understory species were inhibited. As an average of all locations, 65.86% of the plant species were recorded only outside the canopy of *N. glauca*. The vegetation analysis revealed that the SHFA location is more vulnerable to invasion that could be ascribed to its wide range of habitats and high disturbance. The soil–vegetation relationships showed significant variations among the studied locations regarding soil composition, and thereby showed a wide ecological range of the invasive shrubs *N. glauca*. Therefore, the invasion of *N. glauca* in the Taif region altered the species interactions, nutrients, and soil properties.

## 1. Introduction

Invasive species are a serious threat to ecosystems worldwide [1], and they are major drivers of global change. Invasive species threaten habitats and consequently harm the function and structure of ecosystems, either terrestrial or aquatic [2,3]. Invasive species are known to be the main cause of reduction in native species richness and are involved in biodiversity loss, as well as in damage to ecosystem services [4,5]. Subsequently, invasive species lead to a shift in the community structure, thereby increasing the challenge of the conservation of biodiversity and ecosystem functioning worldwide [6]. Researchers’ findings confirm a dramatic decline in native species richness that can ascribed to alien invasions, particularly invasive species from areas with similar climates [7].

Direct impacts of invaders on ecosystem functioning may occur through changes in nutrient levels [4], and they can also change the soil water dynamics and energy budgets, thereby influencing native flora, fauna, and ecosystem services [8,9]. Invasive plants usually come before native plants in the use of nutrient and water resources, thereby colonizing new habitats. This is because most invasive plants have important features like short life cycles, speedy growth, extraordinary reproductive ability, and high competitive efficiency. These characteristics make the invasive plant species superior to native species, and more successful in many habitats [10,11,12,13].

Understanding the invasion dynamics is important for restoring low-resource environments, and it is known that many of these environments may have high species diversity [14], because native species can survive in low-resource environments [15]. It appears that both invasive and native species in low-resource environments are functionally alike [16], suggesting opportunities to restore the invaded plant communities.

Many hypotheses explain plant species interactions during the invasion process. According to the evolution of the increased competitive ability hypothesis, introduced plants can reallocate resources from defense mechanisms to growth and development mechanisms in response to a release from stress [17]. The novel weapons hypothesis proposes that invasive plants have a competitive advantage through their ability to develop allelopathic defenses to compete in invaded habitats via the production of toxic secondary compounds in the environment [18]. High propagule pressure is a key cause of invasion success, according to the propagule pressure hypothesis [19]. The fluctuating resource hypothesis proposes that the plant community becomes more susceptible to invasion whenever there is an increase in the amount of resources that are not totally used [20]. Comprehension of the mechanisms of invasion is essential for creating modern ways to control or manage invasive species, and for prevention or mitigation of the consequences of invasions [21]. However, predicting the ecological behavior of a species in a new environment may be effectively impossible; therefore, it is challenging to predict invasions [22].

Tree tobacco (*Nicotiana glauca* Graham) belongs to the Solanaceae family. It is a native perennial shrub in northwest Bolivia and Argentina, and it is considered an invasive cosmopolitan fast-growing plant in many counties such as Mexico, United States, South Africa, Morocco, Namibia, Egypt, Saudi Arabia, Croatia, and Australia [23,24,25]. Tree tobacco has spread widely worldwide through human activity and naturalization in warm temperate regions [26]. *N. glauca* is listed in the Global Invasive Species Database (GISD) and the Invasive Species Compendium (CABI). *N. glauca* is distributed in a wide range of altitudes and soil conditions, where it can grow rapidly, forming a monospecific stand [26]. It can grow as isolated patches in a broad range of various habitats such as rocky sites, roadsides, arid grasslands, coastal beaches, and any disturbed areas [27]. Moreover, *N. glauca* has few enemies, as it contains anabasine, a chemical compound toxic to humans and animals [28].

Over the past few decades, numerous introduced plants have occupied large areas of Saudi Arabia, especially in the southwest region, which contains 74% of the flora species. *N. glauca* is one of the worst invaders in Saudi Arabia, where it can grow at altitudes of 800–2700 m a.s.l. [29]. However, no study has dealt with the vegetation composition of the habitats invaded by *N. glauca*. We believe that comprehension of the ecological characteristics, distribution, and impacts of *N. glauca* will support appropriate control methods of this deleterious plant and conserve natural vegetation. Hence, the present study aims to evaluate the influence of *N. glauca* invasion on the vegetation composition and soil factors of the invaded locations in the Taif region, Saudi Arabia.

## 2. Results

### 2.1. Floristic Analysis of the Invaded Locations by N. glauca

**Figure 1:**
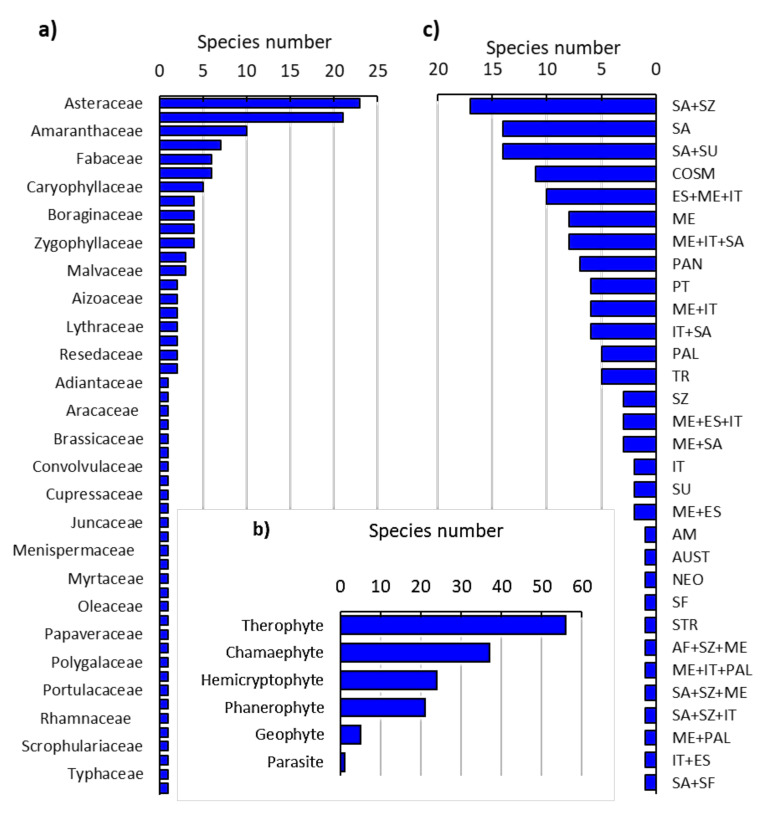
Floristic composition of the habitats invaded by *Nicotiana glauca* in the Taif region, western of Saudi Arabia. (**a**) Identified plant families, (**b**) life forms, and (**c**) chorotype spectra. ME: Mediterranean, COSM: Cosmopolitan, SU: Sudanian, SA: Saharo-Arabian, AM: American, TR.: Tropical, ES: Euro-Siberian, IT: Irano-Turanian, AU: Australian, SZ: Sudano-Zambezian, Pan: Pantropical, PAL: Palaeotropical.

The floristic analysis of the habitats invaded by *N. glauca* revealed the presence of 144 plant species, which are categorized into 79 perennial, 62 annual, and 3 biennial species (Table S1). The identified species belong to 50 families, where Asteraceae, Poaceae, Amaranthaceae, Solanaceae, Fabaceae, Lamiaceae, and Caryophyllaceae were the major families, representing 54.17% of the total species (Figure 1a). These species were distributed over six studied locations that represented three regions (Alwaht (WHT), Ash-shafa (SHFA), and Ar-Ruddaf (RDF)). The richest locations were SHFA1 and SHFA 2, presenting 87 species each. The RDF location presented 77 species, while WHT1 and WHT2 presented 69 and 47 species, respectively.

The recorded plant species can be categorized into six life forms following Raunkiaer’s system, of which 38.89% of the species were therophytes. The other life forms can be sequenced as follows: chamaephyte > hemicryptophyte > phanerophyte > geophyte > parasite (Figure 1b). On the other hand, the chorological analysis of the recorded species revealed that 47.22% of the species were monoregional; specifically, the Saharo-Arabian (SA) element was the main identified chorotype (Figure 1c). On the other hand, 35.42% of the identified species were categorized as bioregional plants, where the Saharo-Arabian-Sudanian (SA-SU) element was the most represented one (9.72%). The pluriregional chorotype was the least represented, accounting for 17.36% of the total recorded species, and the most represented element was the Euro-Siberian (ES) + ME + Irano-Turanian (IT) (6.94%).

### 2.2. Nicotiana glauca Shrub Measurements

**Figure 2:**
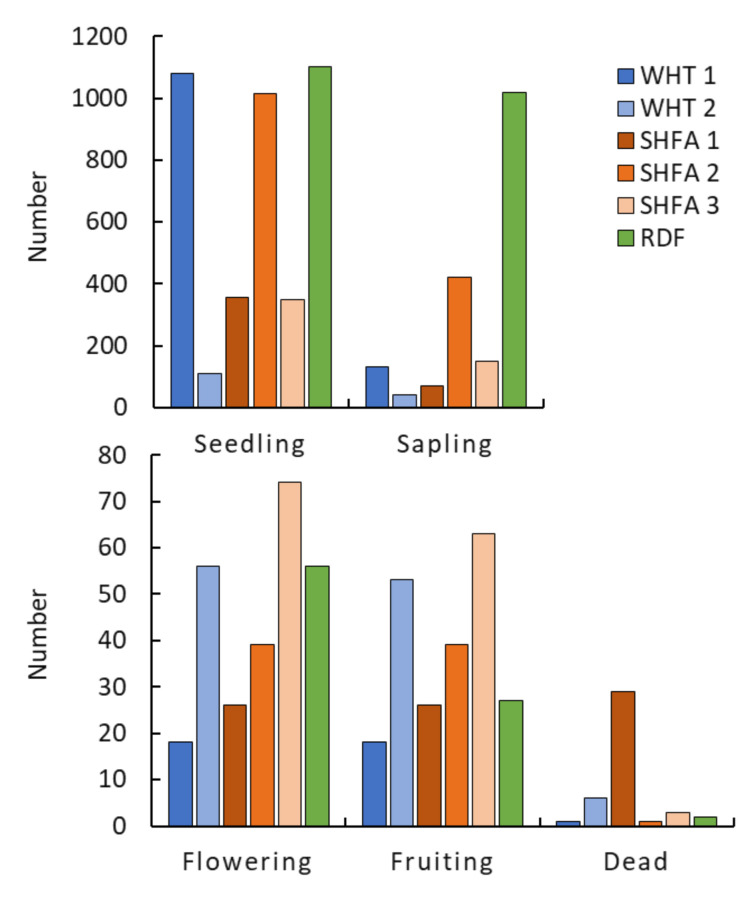
Distribution of *Nicotiana glauca* shrubs by growth stages in different locations in the Taif region, Saudi Arabia. WHT: Alwaht, RDF: Ar-Ruddaf, SHFA: Ash-shafa.

The studied locations showed a substantial variation of the different growth stages of *N. glauca* shrubs (Figure 2). The highest number (1103) of seedlings was reported in the RDF location, while the lowest number (110) of seedlings was recorded in WHT 2 (Figure 2). Additionally, the highest representation of the sapling stage was observed in the RDF location, while the lowest was recorded in WHT 2. Concerning the reproductive stages (flowering and fruiting), the highest number of flowering and fruiting shrubs was recorded in the SHFA 3 location. Meanwhile, the lower number was recorded in WHT 1. Finally, the highest number of dead shrubs was recorded in SHFA 1.

**Figure 3:**
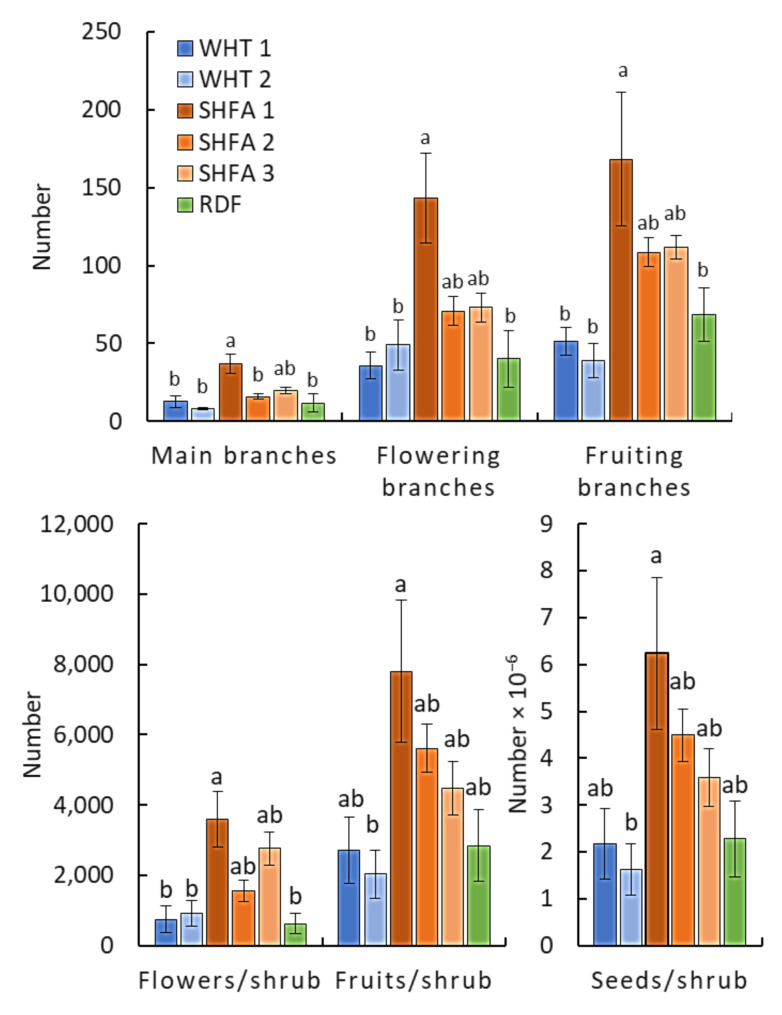
Variation in the different measurements of *Nicotiana glauca* shrubs among the studied locations in the Taif region, Saudi Arabia. WHT: Alwaht, SHFA: Ash-shafa, RDF: Ar-Ruddaf. Different letters for each measurement mean values significant differences at *p* < 0.05 (after Tukey’s HSD).

The shrub measurements of *N. glauca* showed a significant variation among the studied locations (Figure 3). The shrubs growing in SHFA 1 showed the highest number of all measured parameters (main branches, flowering branches, fruiting branches, number of flowers per shrub, number of fruits per shrub, and number of seeds per shrub). However, the other locations did not show significant variation among any of the measured parameters. Overall, it is clear that the SHFA locations showed higher representation of all measured parameters, compared to the WHT and RDF locations.

### 2.3. Vegetation Analysis of the Invaded Locations by N. glauca

The species richness and evenness, as well as the dominant and important species under and outside *N. glauca* canopy, are shown in Table 1. WHT 1 location showed high richness and evenness, under or outside the canopy of *N. glauca*, where 36 species were recorded under the canopy and 28 species were found outside the canopy. *Chenopodium vulvaria* was the most dominant species and *Aizoon canariense* was the second in this location, while *Verbesina encelioides*, *Salsola kali*, *Aristida mutabilis*, and *Tribulus macropterus* were observed as important species with relatively higher importance value based on density and cover under *N. glauca* canopy. The WHT 2 location showed less richness and evenness compared to the WHT 1 location. The most dominant species of this location was *Tribulus macropterus*, while the second most dominant species was *Cynodon dactylon* under *N. glauca* canopy (Table 1). The relative densities of all recorded species are shown in Table S2.

On the other hand, the richness and evenness of the species under the canopy of *N. glauca* were lower than outside the canopy of all studied locations in SHFA. *Cynodon dactylon* dominated all locations of SHFA, either as first or second most dominant, except for the SHFA 2 location. This location was dominated by *Chenopodium murale* as the most dominant and *Poa annua* as the second most dominant species (Table 1). The other important species in this location were *Malva parviflora*, *Verbena officinalis*, *Chenopodium murale*, *Pulicaria arabica*, *Plantago major*, *Euryops arabicus*, *Pulicaria undulata*, *Poa annua*, *Echinops spinosus*, and *Hyparrhenia hirta*. In the same way, the species richness and evenness were higher outside the canopy of *N. glauca* compared to under canopy in the RDF location, where the Simpson’s diversity index and Shannon evenness index outside the canopy attained values of 0.96 and 0.94, respectively, while they were 0.94 and 0.92 under canopy microhabitat (Table 1).

*Aizoon canariense* dominated the RDF location either under or outside the canopy. The other important species under the canopy were *Cynodon dactylon*, *Fagonia bruguieri*, and *Frankenia pulverulenta*, while *Heliotropium curassavicum* and *Fagonia bruguieri* were observed as important species outside *N. glauca* canopy.

### 2.4. Influence of N. glauca on the Native Understory Plant Species

**Figure 4:**
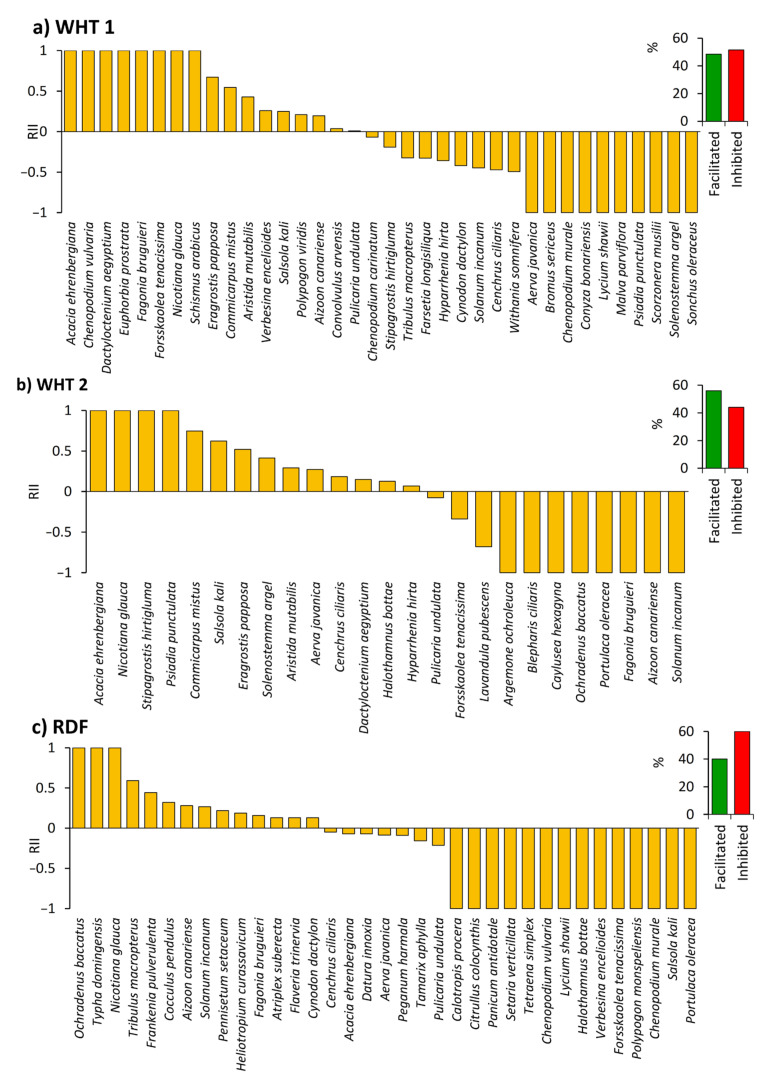
The influence of *Nicotiana glauca* on the native understory species in Alwaht (WHT) (**a**,**b**) and Ar Ruddaf (RDF) (**c**) locations, Taif region, western Saudi Arabia. RII: relative interaction index with ranges from −1 (showing negative effects) to +1 (facilitation). The percentage of facilitated or inhibited species within each location is shown to the right of the histograms.

From the vegetation analysis data, and based on the relative interaction index (RII), there is inconsistency regarding the influence of *N. glauca* on the understory species. In the WHT 1 location, 17 species were facilitated and 18 species were inhibited, and out of these species, 10 species were completely absent outside the canopy (Figure 4a). These species are *Acacia ehrenbergiana*, *Chenopodium vulvaria*, *Dactyloctenium aegyptium*, *Euphorbia prostrata*, *Fagonia bruguieri*, *Forsskaolea tenacissima*, *Nicotiana glauca*, and *Schismus arabicus*. However, *Aerva* javanica, *Bromus sericeus*, *Chenopodium murale*, *Conyza* bonariensis, *Lycium shawii*, *Malva parviflora*, *Psiadia punctulata*, *Scorzonera musilii*, *Solenostemma argel*, and *Sonchus oleraceus* were recorded only outside the canopy of *N. glauca*.

However, in the WHT 2 location, 14 plant species were facilitated and 11 plant species were inhibited (Figure 4b). In the RDF location, 60.0% of the plant species were inhibited and 66.7% of them were completely absent under the canopy of *N. glauca* (Figure 4c). On the other hand, 14 plant species were facilitated and *Ochradenus baccatus*, *Typha domingensis*, and *Nicotiana glauca* were recorded only under the canopy.

**Figure 5:**
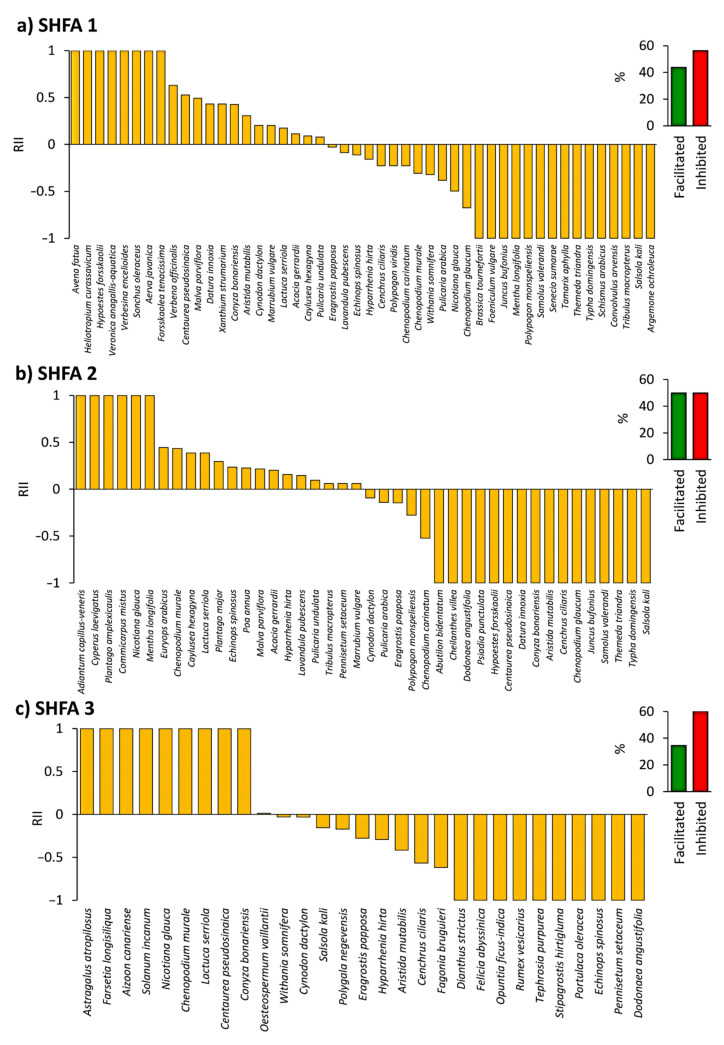
The influence of *Nicotiana glauca* on the native understory species in the Ash-shafa (SHFA) locations [(**a**) SHFA 1, (**b**) SHFA 2 & (**c**) SHFA 3], Taif region, western Saudi Arabia. RII: relative interaction index with ranges from −1 (showing negative effects) to +1 (facilitation). The percentage of facilitated or inhibited species within each location is shown to the right of the histograms.

Regarding the SHFA locations, inconsistency was observed among the three studied locations, where the number of inhibited species was higher than facilitated in SHFA 1 and SHFA 3 locations, while SHFA 2 had equal numbers of facilitated and inhibited species (Figure 5).

**Figure 6:**
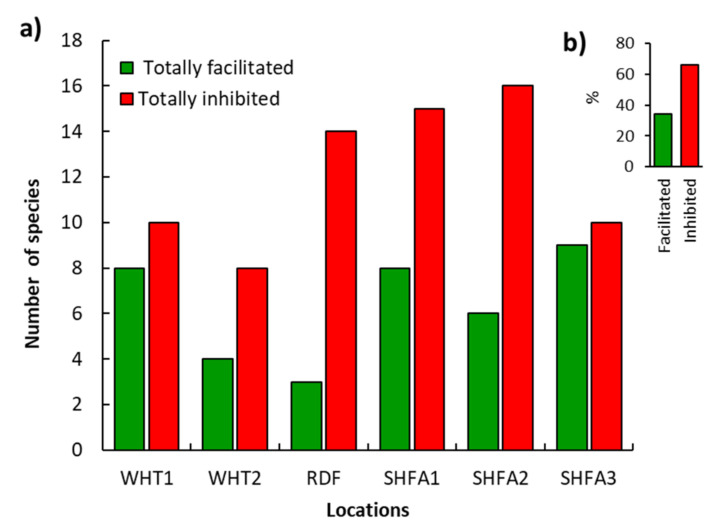
The recorded plant species under the canopy of Nicotiana glauca (facilitated) and outside the canopy (inhibited) in different locations at the Taif region, western Saudi Arabia (**a**), and the average percentage of all locations (**b**).

By calculating the totally inhibited (only recorded outside canopy of *N. glauca*) and totally facilitated (only recorded under canopy) species within each location, it is clear that the number of totally inhibited species was higher than facilitated species (Figure 6a). Additionally, as an average of all locations, 65.86% of the plant species were recorded only outside the canopy of *N. glauca* (Figure 6b).

### 2.5. Vegetation–Soil Relationship

**Figure 7:**
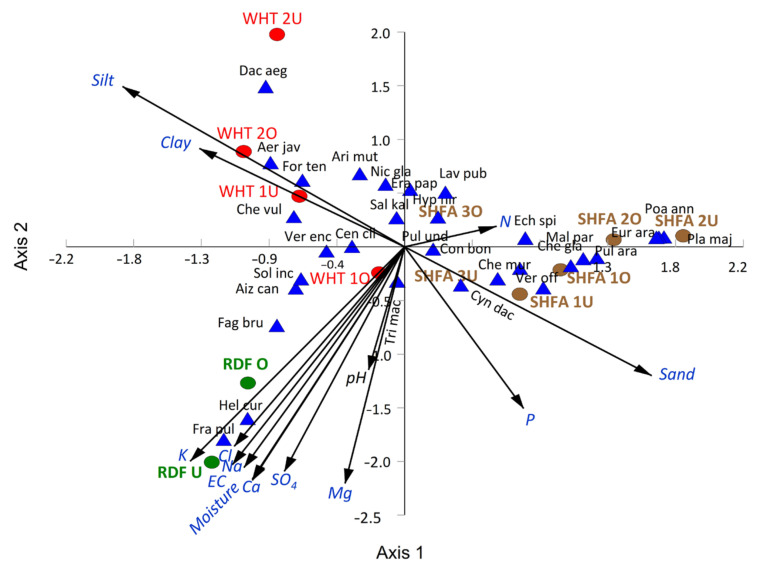
Canonical correspondence analysis (CCA) displaying the correlation between the soil variables and dominant and important species represented the studied locations invaded with *Nicotiana glauca*. The plant species were abbreviated into three-letter of genus and species. WHT: Alwaht, SHFA: Ash-shafa, RDF: Ar Ruddaf, U: under canopy of *N. glauca*, O: outside canopy, EC: electrical conductivity.

The canonical correspondence analysis (CCA) showed that most of the WHT locations were separated on the upper-left side of the CCA biplot, where they are affected by the percentage of silt and clay (Figure 7).

The RDF locations are separated in the lower left side of the CCA biplot, and they show a close correlation to moisture content, salinity, cations (Na^+^, K^+^, Ca^2+^, and Mg^2+^), and anions (SO_4_^2−^ and Cl^−^). Finally, the SHFA locations are segregated on the right side of the CCA biplot, where a correlation to nitrogen, phosphorus, and sand contents can be observed (Figure 7).

**Figure 8:**
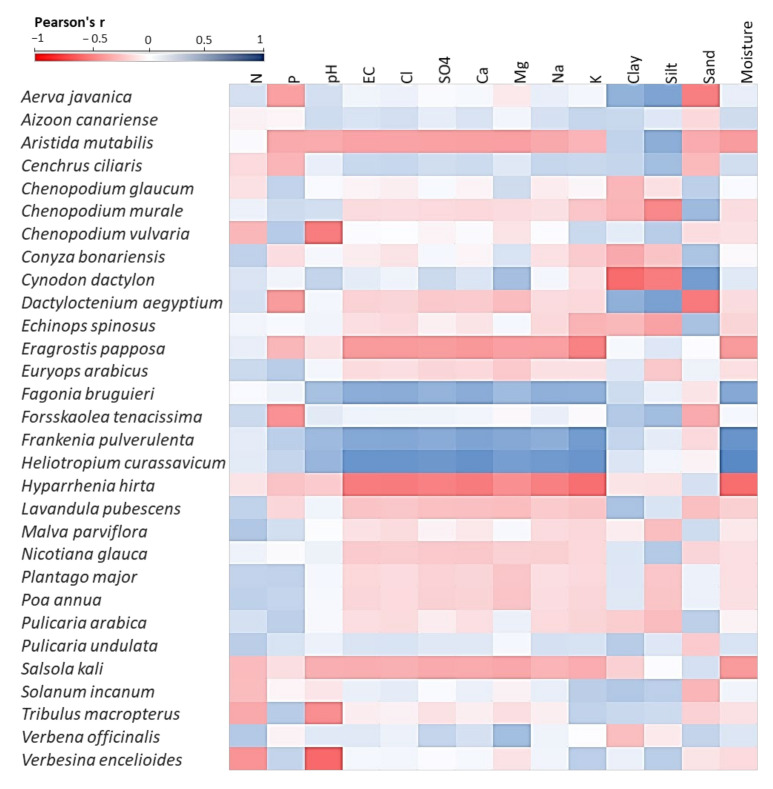
Pearson’s correlation heatmap between the soil variables and the dominant and important species represented in the studied locations invaded with *Nicotiana glauca*.

The Pearson’s correlation analysis between soil variables and dominant and important species is shown in Figure 8. *Cynodon dactylon*, which is the most dominant species of the SHFA 1U, SHFA 1O, SHFA 2O, SHFA 3O locations, and the second most dominant species of the WHT 1O, SHFA 3O, and RDF O locations, showed a positive correlation to all tested soil parameters, except for the contents of clay (r = −0.78) and silt (r = −0.69). On the other hand, *Aizoon canariense*, which is the most dominant species of the WHT 2O, SHFA 3U, RDF U, and RDF O locations, and the second most dominant species of WHT 1U, revealed a positive correlation to all tested parameters, except for sand (r = −0.69), N (r = −0.11) and P (r = −0.09) contents. The grasses, *Aristida mutabilis*, *Eragrostis papposa*, and *Hyparrhenia hirta*, showed significant negative correlations with most of the soil parameters. However, other plants such as *Fagonia bruguieri*, *Frankenia pulverulenta*, *Heliotropium curassavicum* showed a strong positive correlation with most soil parameters.

## 3. Discussion

Invasive plant species are considered one of the most serious problems threatening natural habitats [4,30]. They have been reported to cause biodiversity loss worldwide by hindering the growth and establishment of native species, thereby changing the community assembly [10,31]. In the present study, the floristic analysis of the invaded habitats by *N. glauca* revealed the preponderance of Asteraceae and Poaceae. These families have been reported to be the most represented families in various habitats in Saudi Arabia [32,33]. Additionally, these families were recorded as the main ones in the seed bank under the canopy of *N. glauca* [34]. The perennial species were the most represented in the surveyed locations, although the seed bank had annuals as dominant [34]. This observation showed that *N. glauca* hinders the growth of annuals under their canopies. Invasive plants are known to constrain native species via interference with the resources such as nutrients, soil moisture, and light, as well as usually having allelopathic activity (chemical interference) [10].

According to Raunkiaer’s system, 38.89% of the recorded species were therophytes. This result is in agreement with several studies on the vegetation of Saudi Arabia [32,33,35,36]. Therophytes are characterized by their withstanding dry conditions [37]. Additionally, this life form has a high reproductive capacity, ecological and genetic plasticity [38].

On the other hand, the chorological analysis of the identified species revealed that Saharo-Arabian, Sudano-Zambezian, and cosmopolitan elements were the most represented ones. This showed the wide ecological amplitude and active transport of the studied locations [34,39].

The different stages of *N. glauca* showed significant variation among the studied locations (Figure 2), where the RDF location exhibited the highest number of *N. glauca* seedlings. This observation could be ascribed to the soil seed bank, where the seed bank under the canopy of *N. glauca* growing in the RDF location was rich compared to the other locations [34]. The present results showed that *N. glauca* shrubs growing in SHFA 1 showed the highest number of all measured parameters (main branches, flowering branches, fruiting branches, number of flowers per shrub, number of fruits per shrub, and number of seeds per shrub). It is worth mentioning that this location has various habitats, i.e., roadsides, canal banks, and disturbed areas, indicating that this location is the most vulnerable to invasion by *N. glauca* shrubs. The habitat with low biodiversity and more disturbance has been reported to become more vulnerable to plant invasion [40,41].

The vegetation analysis of the invaded location by *N. glauca* shrubs revealed that the species richness was higher outside the canopy than under canopy (Table 1). The prostrate, perennial grass *Cynodon dactylon* was the dominant or an important species under the canopy of *N. glauca* shrubs in the studied locations. *Cynodon dactylon* is well adapted to various soil types and conditions, and it tolerates drought and salinity [42]. Additionally, this grass is characterized by a high rate of growth, allowing it to compete for space and nutrients [43]. *Cynodon dactylon* has been reported to possess allelopathic interference [44]. All of these features enable this grass to be a good competitor for invasive species.

Based on the relative interaction index, the influence of *N. glauca* on the understory species varied within different invaded locations. In general, most of the understory species were inhibited by the presence of *N. glauca* shrubs, while some species were facilitated (Figure 4 and Figure 5). Invasive trees and shrubs have been reported to have a competitive effect on the understory species within different ecosystems [10,45]. The invasive shrubs can interfere with the understory species either directly or indirectly. They can compete for nutrients, light, and space, as well as they can chemically interact via the production of allelochemicals [11,46]. Additionally, the invasive species modulate the soil composition and soil microflora which in consequence make a shift in the community assembly [47]. *N. glauca* has been reported to possess allelopathic activity against various plants [48,49]. This activity could be ascribed to the bioactive compounds characterized in *N. glauca*, such as anabasine, nicotine, nornicotine, myosmine, scopoletin, and cotinine [50].

On the other hand, some invasive plants have been reported to facilitate the native understory species [51]. In the present study, we observed four invasive species in the studied locations other than *N. glauca*. Among them, *Heliotropium curassavicum* was recorded in more abundance under the canopy of *N. glauca* than outside the canopy, which is known as the invasional meltdown hypothesis. In this hypothesis, the invading species facilitate other exotic species [52]. However, the reverse was observed for the invasive plants *Argemone ochroleuca*, *Opuntia ficus-indica*, and *Datura innoxia*, which were recorded outside the canopy of *N. glauca* only. In the desert ecosystem, invasive shrubs have been reported to facilitate the exotic annuals than native ones [51]. *Argemone ochroleuca* and *Opuntia ficus-indica* have been reported to be among the worst invasive species in Saudi Arabia [29]. Fortunately, the present data showed that *N. glauca* did not facilitate these species. Summing up, some native species benefit from the presence of *N. glauca*, while others are inhibited (Figure 4 and Figure 5). The invasive species can facilitate native species via several mechanisms, either directly through habitat modification or indirectly by facilitating pollination, competitive release, predatory release, and alteration of soil microbial communities [53,54,55]. Furthermore, invasive species can facilitate the native species via modifying the nutrient availability, increasing the organic matter and water in the microhabitat under their canopy [53,56]. Therefore, we can say that this invasive species has a dual effect. This point needs more investigation by studying the long-term effect of *Nicotiana glauca* on the native vegetation.

The CCA showed that most of the WHT locations seem to be correlated with silt and clay percentages, while the SHFA locations exhibited a correlation to N, P, and sand contents. Finally, the RDF locations showed a close correlation to moisture content, salinity, cations, and anions. The correlation of RDF locations to moisture can be ascribed to the nature of this location as a big wadi, which receives a high amount of rainfall. Therefore, this location showed a close correlation with moisture content. In addition, the high content of salt could be attributed to the construction of Ghadir Al Banat dam near RDF locations. 

The CCA analysis revealed significant variations among the studied locations with respect to soil composition, and thereby showed the wide ecological range of the invasive shrubs *N. glauca*. Invasive plants can colonize various habitats, as they are characterized by trait plasticity [57]. Additionally, the invasive plants may modify the soil and the microhabitats under their canopies, this enables them to acquire resources [4]. Therefore, invasive plants have many impacts on the plant community structure through direct and indirect interference with soil chemistry and ecosystem function [58]. They can modify soil via root exudation, allelochemical, mobilization, and solubilization of soil nutrients [59,60]. On the other hand, invasive plants are known to modify the rhizospheric microorganisms, and thereby have a specific pattern of the soil microflora [61]. The microflora can alter and change the soil nutrient cycling [8,62]. During litter decomposition of the invasive species in the Mediterranean ecosystem, nitrogen is released at a higher rate compared to native plants. Therefore, they can change the N cycle and promote the shifting of plant assemblages [59].

Specifically, Bermuda grass (*Cynodon dactylon*), which is a dominant or important species in most of the studied locations, showed a positive correlation with most studied soil parameters. This grass has been reported to be well adapted plants to various soil types and conditions, tolerates drought and salinity [42], and has a high rate of growth enabling it to compete for space and nutrients [43]. The grasses, *Aristida mutabilis*, *Eragrostis papposa*, and *Hyparrhenia hirta*, showed a strong negative correlation with moisture content and salinity, as these grasses prefer to grow in sandy habitats with low salinity [63]. *Aristida* species have been reported to colonize poor and dry poor soils in arid habitats [64].

## 4. Materials and Methods

### 4.1. Study Area

**Figure 9:**
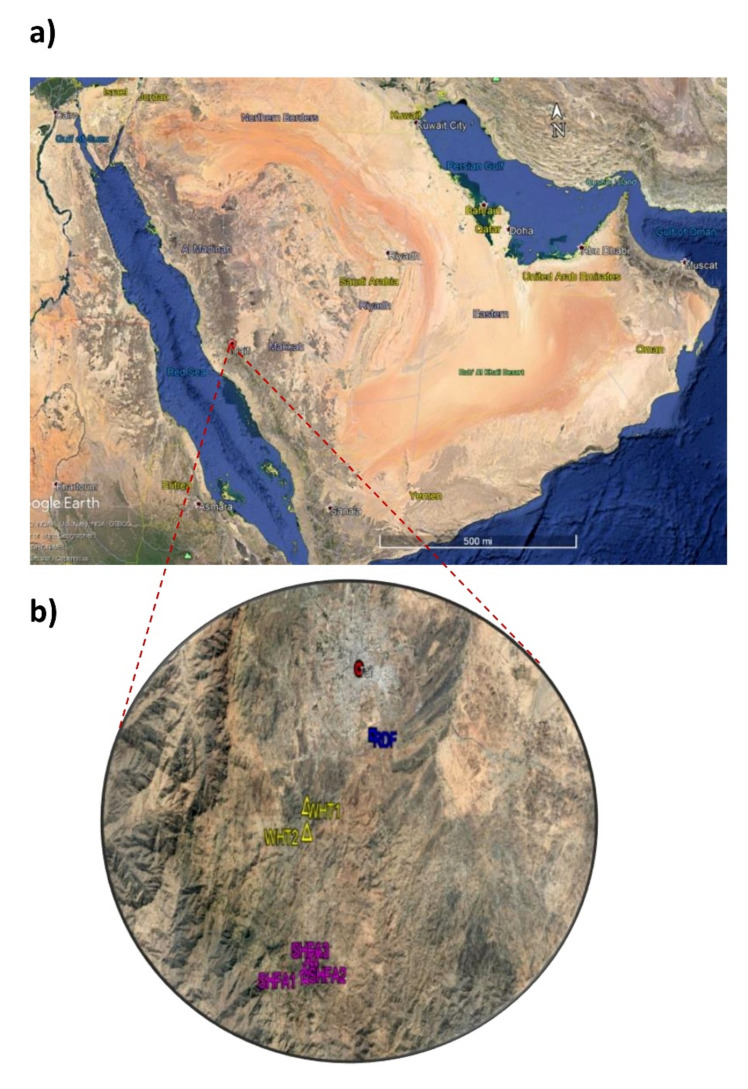
Map of Saudi Arabia displaying the study area location (**a**), and different studied sites (**b**) (Alwaht (WHT), Ash-shafa (SHFA), and Ar Ruddaf (RDF)) in the Taif region.

The studied locations were located in the Taif Governorate, in the western part of Saudi Arabia (Figure 9), between 20–22° N and 40–42° E, occupying about 321 km^2^ in the northern end of the Al-Hijaz Mountains at an altitude of 1700 m a.s.l. [65]. The Taif region is characterized by specific landscapes and fertile soil, where agriculture is an important economic income, such as the cultivation of wheat, lemon, apricot, peaches, pomegranate, grapes, and almonds. The climate of the Taif region is tropical and arid, temperatures are not very hot in summer similar to the lower regions of Saudi Arabia.

Climatic data recorded during 2005–2015 at the Taif Meteorological Station indicated that the range monthly means of the minimum air temperatures were 9–24 °C, and maximum air temperatures were 24–36 °C [65]. The hottest month was August (30 °C), while the coldest month was December (16 °C). The mean monthly humidity was 37.5%. Climatic data show that the annual precipitation of the Taif region was 132.1 mm, while the monthly amount of precipitation was ranges from 1.9 mm in February to 33.2 mm in May (Figure S1).

### 4.2. Studied Locations and Nicotiana glauca Shrub Measurements

Within the Taif region, three sites are selected as most invaded areas namely: Alwaht (WHT), Ash-shafa (SHFA), and Ar-Ruddaf (RDF). A total of six locations were chosen: WHT1, WHT2, SHFA1, SHFA2, SHFA3, and RDF (Figure 9, Table S3). These locations were chosen to cover and represent the studied regions. In May 2020, three *N. glauca* shrubs were selected for measurements in each location and we have taken photographs for each shrub, with a scale (100 cm) in the background, near the trunk. Shrub height (TH), crown diameter (CD), canopy height (CH), trunk diameter (TD), and trunk height (TH) were determined using ImageJ software [66].

On the other hand, the main branches, flowering branches, and fruiting branches of each shrub were counted. From each shrub, three flowering branches were taken randomly, and the flowers were counted, then the average was calculated and multiplied by the total number of flowering branches, to find out the approximate total number of flowers per shrub. Additionally, for the total number of fruits in the shrub, three fruiting branches were taken, and the fruits were counted in each branch, then the average was calculated and multiplied by the total number of fruiting branches. The seeds were counted in 20 fruits, then the average was calculated and multiplied by the total number of fruits in each tree.

In addition, five stands were selected in each location and within each stand, three quadrats (10 × 10 m) were plotted. In each quadrate, the number of seedlings, saplings, mature, fruiting, flowering, and dead *N. glauca* shrubs was counted.

### 4.3. Vegetation Analysis of the Invaded Locations

In each location, five stands were made, within each stand, three quadrats (4 m^2^, each) were plotted under the canopy of N. glauca and other three quadrats outside the canopy. In each quadrat, the plant species were recorded and the density of each species was determined according to Bonham [67]. The nomenclature of the taxa was based on Collenette [33] and Chaudhary [68]. The life forms of each species were identified according to Raunkiaer [69]. The geographical range of the species was obtained perusing available sources from the floras and other published works [70,71,72].

### 4.4. Soil Analysis

From each quadrate, a soil sample was collected, under and outside the canopy of *N. glauca*, at 10–30 cm depth in polythene bags, labeled, and brought shortly to the laboratory in the College of Food and Agricultural Sciences of King Saud University, Kingdom of Saudi Arabia. Additionally, another soil sample was collected in a moisture tin for the determination of soil moisture content by a weight-loss method. Soil samples were spread over paper sheets, air-dried at room temperature, and passed through a 2 mm sieve to remove any foreign materials like wood, rocks, and leaves. The soil texture was determined according to Bouyoucos [73]. Soil–water extracts (1:5) were prepared for assessment of pH and electrical conductivity (EC) [74]. The concentrations of Ca, Mg, Na, and K, were measured using a flame photometer following the methods of Rhoades [75]. Chloride content was determined via titration method using AgNO_3_ [76], while sulphate content was determined gravimetrically by precipitation method using BaCl_2_ [77]. Available phosphorus was quantified calorimetrically as described by Nelson and Sommers [78], while available nitrogen was determined by the Kjeldahl method [79].

### 4.5. Data Treatments

To compare the difference among the six studied locations, the data of shrub measurements were subjected to one-way ANOVA followed by Tukey’s HSD test at a probability level of 0.05 using CoStat 6.3 program (CoHort Software, Monterey, CA, USA). Additionally, the data of diversity indexes (Simpson index and Shannon-evenness) were subjected to one-way ANOVA followed by Tukey’s HSD test. For vegetation analysis data, the relative interaction index (RII) was calculated upon the data of relative densities of the plant species, to assess the effect of *N. glauca* on the biodiversity of the invaded locations as follows:

(1) $$RII=\frac{\left(Species\;diversity_{under\;canopy}-Species\;diversity_{outside\;canopy}\right)}{\left(Species\;diversity_{under\;canopy}+Species\;diversity_{outside\;canopy}\right)}$$

The RII value ranges from −1 (indicating negative effects) to +1 (facilitation), which denotes the intensity of decrease or increase in species diversity [80].

To assess the correlations between the soil variables and dominant and important species presented in the studied locations invaded with *N. glauca*, canonical correspondence analysis (CCA) was conducted according to ter Braak and Schaffers [81]. Two data sets of the plant relative densities and the soil parameters of each quadrate were performed and subjected to CCA was performed using CANOCO software version 4.5 (Biometris, Plant Research International, Wageningen, The Netherlands). Additionally, Pearson’s correlation heatmap between the soil variables and the dominant and important species was generated using the XLSTAT software program (version 2018, Addinsoft, New York, NY, USA).

## 5. Conclusions

The locations invaded by *N. glauca* shrubs showed substantial variation in the community composition, and the influence on the understory species ranged from competition to facilitation, while most species were inhibited. *N. glauca* may facilitate some of the native species via habitat modification. The vegetation analysis showed that SHFA location is more vulnerable to invasion by *N. glauca* shrubs, which can be attributed to the high disturbance in this location as well as its diverse habitats. The soil composition of the studied location showed substantial variation, providing a wide range for *N. glauca* invasion. Hence, the invasion of *N. glauca* shrubs in the Taif region showed alteration in the species interactions as well as nutrients and soil properties. To clarify the dual effect (inhibition or facilitation) by *N. glauca* shrubs in the Taif region, further study is recommended for the evaluation of long-term effects. Additionally, further study is needed to explore the soil microflora of the invaded locations concerning the promoted or inhibited effects on the invasive shrubs and their understory species.

## Figures and Tables

**Table 1:** Plant species richness, evenness, and dominance of the studied locations invaded by *Nicotiana glauca* in the Taif region, western Saudi Arabia.

Location		Total Species no.	Richness (Simpson Index)	Evenness (Shannon-Evenness)	Most Dominant	Second Most Dominant	Important Species
WHT 1	U	36	0.96 ^ab^	0.94 ^b^	*Chenopodium vulvaria* (8.93 *)	*Aizoon canariense* (8.33)	*Verbesina encelioides* (7.54) *Salsola kali* (7.38) *Aristida mutabilis* (6.80) *Tribulus macropterus* (6.19)
	O	28	0.96 ^ab^	0.95 ^b^	*Tribulus macropterus* (12.11)	*Cynodon dactylon* (7.00)	*Cenchrus ciliaris* (6.61) *Aizoon canariense* (5.60) *Hyparrhenia hirta* (4.67) *Solanum incanum* (4.76)
WHT 2	U	17	0.93 ^de^	0.92 ^c^	*Aerva javanica* (16.80)	*Nicotiana glauca* (10.35)	*Eragrostis papposa* (9.90) *Dactyloctenium aegyptium* (9.00) *Aristida mutabilis* (8.70)
	O	21	0.92 ef	0.89 ^e^	*Aizoon canariense* (18.78)	*Forsskaolea tenacissima* (10.85)	*Aerva javanica* (9.62) *Lavandula pubescens* (9.39) *Dactyloctenium aegyptium* (6.68)
SHFA 1	U	33	0.91 ^fg^	0.86 ^g^	*Cynodon dactylon* (26.74)	*Conyza bonariensis* (7.43)	*Malva parviflora* (4.96) *Verbena officinalis* (4.96)
	O	40	0.95 ^bc^	0.89 ^ef^	*Cynodon dactylon* (17.67)	*Chenopodium glaucum* (7.31)	*Chenopodium murale* (5.34) *Pulicaria arabica* (5.27) *Nicotiana glauca* (4.22)
SHFA 2	U	26	0.96 ^a^	0.95 ^b^	*Chenopodium murale* (9.18)	*Poa annua* (7.65)	*Cynodon dactylon* (7.04) *Plantago major* (7.10) *Euryops arabicus* (6.28) *Pulicaria undulata* (6.24)
	O	36	0.97 ^a^	0.97 ^a^	*Cynodon dactylon* (8.49)	*Pulicaria undulata* (5.16)	*Poa annua* (4.83) *Pulicaria arabica* (4.34)
SHFA 3	U	19	0.90 ^g^	0.87 ^f^	*Aizoon canariense* (19.36)	*Cynodon dactylon* (18.72)	*Chenopodium murale* (10.65) *Salsola kali* (6.99) *Nicotiana glauca* (6.29)
	O	20	0.92 ^ef^	0.90 ^d^	*Cynodon dactylon* (19.99)	*Eragrostis papposa* (10.50)	*Salsola kali* (9.58) *Echinops spinosus* (7.76) *Hyparrhenia hirta* (7.30)
RDF	U	21	0.94 ^cd^	0.92 ^c^	*Aizoon canariense* (15.58)	*Heliotropium curassavicum* (10.38)	*Cynodon dactylon* (9.71) *Fagonia bruguieri* (8.26) *Frankenia pulverulenta* (7.08)
	O	32	0.96 ^ab^	0.94 ^b^	*Aizoon canariense* (8.73)	*Cynodon dactylon* (7.49)	*Heliotropium curassavicum* (7.10) *Fagonia bruguieri* (6.00)
*F* value _0.05_			22.26 ***	65.54 ***			

* Values are the importance value based on the relative density of species. WHT: Alwaht, RDF: Ar-Ruddaf, SHFA: Ash-shafa. U: under canopy, O: outside canopy. Different letters among location means significant varation at *p* = 0.05, ns: non-significant, *** significant at *p* < 0.001.

## Data Availability

Samples of the compounds are not available from the authors.

## Supplementary Materials

The following are available online at https://www.mdpi.com/article/10.3390/plants10122587/s1, Figure S1: The data are long-term averages, based on weather reports during 2005–2015, recorded at the Taif meteorological station, Table S1: Floristic analysis of the studied location invaded by *Nicotiana glauca* in Taif region, western Saudi Arabia, Table S2: Vegetation composition of the studied locations invaded by *Nicotiana glauca* in Taif region, western Saudi Arabia, Table S3: Coordinates and altitudes of different locations invaded by *Nicotiana glauca* in Taif region, western Saudi Arabia.

## References

[1] Van Wilgen B., Le Maitre D.C., Cowling R. (1998). Ecosystem services, efficiency, sustainability and equity: South Africa’s Working for Water programme. Trends Ecol. Evol..

[2] Keller R.P., Geist J., Jeschke J.M., Kühn I. (2011). Invasive species in Europe: Ecology, status, and policy. Environ. Sci. Eur..

[3] Pejchar L., Mooney H.A. (2009). Invasive species, ecosystem services and human well-being. Trends Ecol. Evol..

[4] Linders T.E.W., Schaffner U., Eschen R., Abebe A., Choge S.K., Nigatu L., Mbaabu P.R., Shiferaw H., Allan E. (2019). Direct and indirect effects of invasive species: Biodiversity loss is a major mechanism by which an invasive tree affects ecosystem functioning. J. Ecol..

[5] Duenas M.-A., Ruffhead H.J., Wakefield N.H., Roberts P.D., Hemming D.J., Diaz-Soltero H.J.B. (2018). The role played by invasive species in interactions with endangered and threatened species in the United States: A systematic review. Biodivers. Conserv..

[6] Brooks M.L., D’antonio C.M., Richardson D.M., Grace J.B., Keeley J.E., DiTomaso J.M., Hobbs R.J., Pellant M., Pyke D. (2004). Effects of invasive alien plants on fire regimes. BioScience.

[7] Gaertner M., Den Breeyen A., Cang H., Richardson D.M. (2009). Impacts of alien plant invasions on species richness in Mediterranean-type ecosystems: A meta-analysis. Prog. Phys. Geogr..

[8] Liao C., Peng R., Luo Y., Zhou X., Wu X., Fang C., Chen J., Li B. (2008). Altered ecosystem carbon and nitrogen cycles by plant invasion: A meta-analysis. New Phytol..

[9] Hulme P., Pyšek P., Jarošík V., Pergl J., Schaffner U., Vila M. (2013). Bias and error in current knowledge of plant invasions impacts. Trends Ecol. Evol..

[10] Abd El Gawad A., El-Amier Y. (2015). Allelopathy and potential impact of invasive *Acacia saligna* (Labill.) Wendl. on plant diversity in the Nile Delta coast of Egypt. Int. J. Environ. Res..

[11] Bonanomi G., Incerti G., Abd El-Gawad A.M., Sarker T.C., Stinca A., Motti R., Cesarano G., Teobaldelli M., Saulino L., Cona F. (2018). Windstorm disturbance triggers multiple species invasion in an urban Mediterranean forest. iFor. Biogeosci. For..

[12] Wang Y.-J., Chen D., Yan R., Yu F.-H., van Kleunen M. (2019). Invasive alien clonal plants are competitively superior over co-occurring native clonal plants. Perspect. Plant Ecol. Evol. Syst..

[13] Rai P.K., Singh J. (2020). Invasive alien plant species: Their impact on environment, ecosystem services and human health. Ecol. Indic..

[14] Weigelt A., Weisser W.W., Buchmann N., Scherer-Lorenzen M. (2009). Biodiversity for multifunctional grasslands: Equal productivity in high-diversity low-input and low-diversity high-input systems. Biogeosciences.

[15] Daehler C.C. (2003). Performance comparisons of co-occurring native and alien invasive plants: Implications for conservation and restoration. Ann. Rev. Ecol. Evol. Syst..

[16] Funk J.L., Standish R.J., Stock W.D., Valladares F. (2016). Plant functional traits of dominant native and invasive species in mediterranean-climate ecosystems. Ecol. Lett..

[17] Blossey B., Notzold R. (1995). Evolution of increased competitive ability in invasive nonindigenous plants: A hypothesis. J. Ecol..

[18] Callaway R.M., Ridenour W.M. (2004). Novel weapons: Invasive success and the evolution of increased competitive ability. Front. Ecol. Environ..

[19] Lockwood J.L., Cassey P., Blackburn T. (2005). The role of propagule pressure in explaining species invasions. Trends Ecol. Evol..

[20] Davis M.A., Grime J.P., Thompson K. (2000). Fluctuating resources in plant communities: A general theory of invasibility. J. Ecol..

[21] Petruzzella A., Tauany A., van Leeuwen C.H., de Assis Esteves F., Figueiredo-Barros M.P., Bakker E.S. (2020). Species identity and diversity effects on invasion resistance of tropical freshwater plant communities. Sci. Rep..

[22] Williamson M. (1999). Invasions. Ecography.

[23] Issaly E.A., Sérsic A.N., Pauw A., Cocucci A.A., Traveset A., Benitez-Vieyra S.M., Paiaro V. (2020). Reproductive ecology of the bird-pollinated Nicotiana glauca across native and introduced ranges with contrasting pollination environments. Biol. Invasions.

[24] Bogdanović S., Mitić B., Ruščić M., Dolina K. (2006). *Nicotiana glauca* Graham (Solanaceae), a new invasive plant in Croatia. Acta Bot. Croat..

[25] Schueller S.K. (2004). Self-pollination in island and mainland populations of the introduced hummingbird-pollinated plant, *Nicotiana glauca* (Solanaceae). Am. J. Bot..

[26] Florentine S., Westbrooke M., Gosney K., Ambrose G., O’Keefe M. (2006). The arid land invasive weed *Nicotiana glauca* R. Graham (Solanaceae): Population and soil seed bank dynamics, seed germination patterns and seedling response to flood and drought. J. Arid Environ..

[27] Nattero J., Cocucci A.A. (2007). Geographical variation in floral traits of the tree tobacco in relation to its hummingbird pollinator fauna. Biol. J. Linn. Soc..

[28] Scharenberg F., Stegemann T., Çiçek S.S., Zidorn C. (2019). Sequestration of pyridine alkaloids anabasine and nicotine from *Nicotiana* (Solanaceae) by *Orobanche ramosa* (Orobanchaceae). Biochem. Syst. Ecol..

[29] Thomas J., El-Sheikh M.A., Alfarhan A.H., Alatar A.A., Sivadasan M., Basahi M., Al-Obaid S., Rajakrishnan R. (2016). Impact of alien invasive species on habitats and species richness in Saudi Arabia. J. Arid Environ..

[30] Didham R.K., Tylianakis J.M., Hutchison M.A., Ewers R.M., Gemmell N.J. (2005). Are invasive species the drivers of ecological change?. Trends Eco. Evol..

[31] Weidlich E.W., Flórido F.G., Sorrini T.B., Brancalion P.H. (2020). Controlling invasive plant species in ecological restoration: A global review. J. Appl. Ecol..

[32] Abdel Khalik K., Al-Gohary I., Al-Sodany Y. (2017). Floristic composition and vegetation: Environmental relationships of Wadi Fatimah, Mecca, Saudi Arabia. Arid Land Res. Manag..

[33] Collenette S. (1999). Wildflowers of Saudi Arabia.

[34] Alharthi A.S., Abd-ElGawad A.M., Assaeed A.M. (2021). Influence of the invasive shrub *Nicotiana glauca* Graham on the plant seed bank in various locations in Taif region, western of Saudi Arabia. Saudi J. Biol. Sci..

[35] Khalik K.A., El-Sheikh M., El-Aidarous A. (2013). Floristic diversity and vegetation analysis of wadi Al-Noman, Mecca, Saudi Arabia. Turk. J. Bot..

[36] Osman A.K., Al-Ghamdi F., Bawadekji A. (2014). Floristic diversity and vegetation analysis of Wadi Arar: A typical desert Wadi of the Northern Border region of Saudi Arabia. Saudi J. Biol. Sci..

[37] Tarhouni M., Ben Hmida W., Neffati M. (2017). Long-term changes in plant life forms as a consequence of grazing exclusion under arid climatic conditions. Land Degrad. Dev..

[38] Abd El-Gawad A.M. (2014). Ecology and allelopathic control of *Brassica tournefortii* in reclaimed areas of the Nile Delta, Egypt. Turk. J. Bot..

[39] Alsherif E.A., Fadl M.A. (2016). Floristic study of the Al-shafa highlands in Taif, Western Saudi Arabia. Flora.

[40] Wan J.-Z., Zhang Z.-X., Wang C.-J. (2019). Effects of ecoregional vulnerability on habitat suitability of invasive alien plants: An assessment using 13 species on a global scale. Environ. Earth Sci..

[41] Liao H., Luo W., Peng S., Callaway R.M. (2015). Plant diversity, soil biota and resistance to exotic invasion. Divers. Distrib..

[42] Shi H., Ye T., Chan Z. (2013). Comparative proteomic and physiological analyses reveal the protective effect of exogenous polyamines in the bermudagrass (*Cynodon dactylon*) response to salt and drought stresses. J. Proteome Res..

[43] Juraimi A.S., Drennan S.D., Anuar N. (2005). Competitive effect of *Cynodon dactylon* (L.) Pers. on four crop species, soybean [*Glycine max* (L.) Merr.], maize (*Zea mays*), spring wheat (*Triticum aestivum*) and faba bean [*Vicia faba* (L.)]. Asian J. Plant Sci..

[44] Liang Z., Li X., Zhang H., Li J., Bian X., Xu J. (2018). Allelopathic effects of Bermuda grass (*Cynodon dactylon* L.) root exudates on seed germination and seedling growth of tall fescue (*Festuca arundinacea* Schreb). Allelopathy J..

[45] Ward J.S., Williams S.C., Linske M.A. (2018). Influence of invasive shrubs and deer browsing on regeneration in temperate deciduous forests. Can. J. For. Res..

[46] Medina-Villar S., Alonso Á., Castro-Díez P., Pérez-Corona M.E. (2017). Allelopathic potentials of exotic invasive and native trees over coexisting understory species: The soil as modulator. Plant Ecol..

[47] Woodworth G.R., Ward J.N., Carr D.E. (2020). Exotic tree and shrub invasions alter leaf-litter microflora and arthropod communities. Oecologia.

[48] Alshahrani S.T. (2008). Effect of aqueous extract of the invasive species tobacco (*Nicotiana glauca* L.) on seedlings growth of Juniper (*Juniperus procera* L.). Emir. J. Food Agric..

[49] El-Kenany E.T., El-Darier S.M., Abdellatif A.A., Shaklol S.M. (2017). Allelopathic potential of invasive species: *Nicotiana glauca* Graham on some ecological and physiological aspects of *Medicago sativa* L. and *Triticum aestivum* L. Rend. Lincei.

[50] Kasiotis K.M., Evergetis E., Papachristos D., Vangelatou O., Antonatos S., Milonas P., Haroutounian S.A., Machera K. (2020). An essay on ecosystem availability of *Nicotiana glauca* graham alkaloids: The honeybees case study. BMC Ecol..

[51] Lucero J.E., Noble T., Haas S., Westphal M., Butterfield H.S., Lortie C.J. (2019). The dark side of facilitation: Native shrubs facilitate exotic annuals more strongly than native annuals. NeoBiota.

[52] Green P.T., O’Dowd D.J., Abbott K.L., Jeffery M., Retallick K., Mac Nally R. (2011). Invasional meltdown: Invader-invader mutualism facilitates a secondary invasion. Ecology.

[53] Rodriguez L.F. (2006). Can invasive species facilitate native species? Evidence of how, when, and why these impacts occur. Biol. Invasions.

[54] Wundrow E.J., Carrillo J., Gabler C.A., Horn K.C., Siemann E. (2012). Facilitation and competition among invasive plants: A field experiment with alligatorweed and water hyacinth. PLoS ONE.

[55] Zhang Z., Liu Y., Brunel C., van Kleunen M. (2020). Soil-microorganism-mediated invasional meltdown in plants. Nat. Ecol. Evol..

[56] Shumway S.W. (2000). Facilitative effects of a sand dune shrub on species growing beneath the shrub canopy. Oecologia.

[57] Assaeed A.M., Al-Rowaily S.L., El-Bana M.I., Hegazy A.K., Dar B.A., Abd-ElGawad A.M. (2020). Functional traits plasticity of the invasive herb *Argemone ochroleuca* Sweet in different arid habitats. Plants.

[58] Milanović M., Knapp S., Pyšek P., Kühn I. (2020). Linking traits of invasive plants with ecosystem services and disservices. Ecosyst. Serv..

[59] Incerti G., Cartenì F., Cesarano G., Sarker T.C., El-Gawad A., Ahmed M., D’Ascoli R., Bonanomi G., Giannino F. (2018). Faster N release, but not C loss, from leaf litter of invasives compared to native species in Mediterranean ecosystems. Front. Plant Sci..

[60] Weidenhamer J.D., Callaway R.M. (2010). Direct and indirect effects of invasive plants on soil chemistry and ecosystem function. J. Chem. Ecol..

[61] Rout M.E., Callaway R.M. (2012). Interactions between exotic invasive plants and soil microbes in the rhizosphere suggest that ‘everything is not everywhere’. Ann. Bot..

[62] Hawkes C.V., Wren I.F., Herman D.J., Firestone M.K. (2005). Plant invasion alters nitrogen cycling by modifying the soil nitrifying community. Ecol. Lett..

[63] McCormick L., McMillan M., Lodge G. (1992). Coolatai grass (*Hyparrhenia hirta*) control. Aust. Weeds Res. Newsl..

[64] Chaudhary S.A. (1989). Grasses of Saudi Arabia.

[65] Vincent P. (2008). Saudi Arabia: An Environmental Overview.

[66] Schindelin J., Arganda-Carreras I., Frise E., Kaynig V., Longair M., Pietzsch T., Preibisch S., Rueden C., Saalfeld S., Schmid B. (2012). Fiji: An open-source platform for biological-image analysis. Nat. Methods.

[67] Bonham C.D. (2013). Measurements for Terrestrial Vegetation.

[68] Chaudhary S.A. (1999). Flora of the Kingdom of Saudi Arabia.

[69] Raunkiaer C. (1937). Plant Life Forms.

[70] Zohary M. (1973). Geobotanical Foundations of the Middle East.

[71] Fadl M.A., Farrag H.F., Al-Sherif E. (2015). Floristic composition and vegetation analysis of wild legumes in Taif district, Saudi Arabia. Int. Res. J. Agric. Sci. Soil Sci..

[72] Farrag H.F. (2012). Floristic composition and vegetation-soil relationships in Wadi Al-Argy of Taif region, Saudi Arabia. Int. Res. J. Plant Sci..

[73] Bouyoucos G.J. (1962). Hydrometer method improved for making particle size analyses of soils. Agron. J..

[74] Rowell D. (1994). Soil Science: Method and Applications.

[75] Rhoades J., Page A.L., Miller R.H., Keeney D.R. (1982). Soluble salts. Methods of Soil Analysis: Part 2: Chemical and Microbiological Properties.

[76] Jackson M.L. (1962). Soil Chemical Analysis.

[77] Piper C.S. (1947). Soil and Plant Analysis.

[78] Nelson D., Sommers L., Page A.L. (1982). Chemical and microbiological properties. Methods for Soil Analysis.

[79] Bremner J., Mulvaney C., Page A.L., Miller R.H., Keeney D.R. (1982). Total nitrogen. Methods of Soil Analysis.

[80] Armas C., Ordiales R., Pugnaire F.I. (2004). Measuring plant interactions: A new comparative index. Ecology.

[81] Ter Braak C.J., Schaffers A.P. (2004). Co-correspondence analysis: A new ordination method to relate two community compositions. Ecology.

