# Supplementary material for: Impacts of Nicotiana glauca Graham Invasion on the Vegetation Composition and Soil: A Case Study of Taif, Western Saudi Arabia
Source: Plants (Basel). 2021 Nov 25;10(12):2587. doi: 10.3390/plants10122587 (PMC8708854; doi:10.3390/plants10122587)
Supplement: Supplementary file 1 [file plants-10-02587-s001.zip › plants-1425979-supplementary.pdf]

Table S1. Floristic analsis of the studied location invaded by *Nicotiana glauca* in Taif region, western of Saudi Arabia.

Family	Botanical name	Chorotype	Life span	Life form	WHT 1	WHT2	SHFA1	SHFA2	SHFA3	RDF
Acanthaceae	*Blepharis attenuata* Napper.	SA+SU+IT	Per.	Ch	√	√				
	Hypoestes forsskaolii (Vahl) R.Br.	SZ	Per.	Ch			√	√	√	
Adiantaceae	*Cheilanthes vellea* (Aiton) Domin	ME	Ann.	Ge				√		
Aizoaceae	*Aizoon canariense* L.	SA+SZ	Ann.	Th	√	√	√	√	√	√
	Carpobrotus edulis (L.) N.E.Br.	SF	Per.	Ch						√
Amaranthaceae	*Aerva javanica* (Burm.f.) Juss. ex Schultes	SA+TR	Per.	Ch	√	√		√	√	√
	Atriplex leucoclada Boiss.	IT+SA+SU	Per.	Ch						√
	Chenopodium carinatum R.Br.	SA+SZ	Ann.	Th	√		√	√	√	√
	Chenopodium glaucum L.	ME+ES	Ann.	Th	√		√	√		√
	Chenopodium murale L.	COSM	Ann.	Th	√		√	√	√	√
	Chenopodium vulvaria L.	COSM	Ann.	Th			√	√		√
	Chenopodium album L.	COSM	Ann.	Th			√	√		√
	Halothamnus bottae Jaub. & Spach.	SA	Per.	Ch		√				√
	Salsola arabica Botsch.	SA+SU	Per.	Ch	√					√
	Salsola kali L.	PT	Ann.	Th	√	√	√	√	√	√
Apocynaceae	*Solenostemma argel* (Del.) Hayne	SA	Per.	Ch	√	√				√
Aracaceae	*Phoenix caespitosa* Chiov.	SU+ZA+SA	Per.	Ph			√			
Asclepiadaceae	*Calotropis procera* (Ait.) Ait.f.	SU+ZA+SA	Per.	Ph	√	√			√	√
	Caralluma tuberculata N.E. Br.	SA+SU	Per.	He		√				
	Pergularia tomentosa L.	SA+SU	Per.	Ch						√
	Periploca aphylla Decne	SA+SU	Per.	Ch	√	√			√	√
Asphodelaceae	*Asphodelus tenuifolius* Cav.	SA+SU	Ann.	Th	√				√	
Brassicaceae	*Farsetia longisiliqua* Decne.	SU+ZA+SA	Per.	Ch	√					
	Brassica tournefortii Guan.	ME+SA	Ann.	Th		√	√	√		
Boraginaceae	*Echium angustifolium* Mill.	ME	Per.	Ch						√
	Heliotropium curassavicum L.	NEO	Per.	Ch	√		√	√		√
	Lepidium didymum L.	COSM	Bi.	Th			√	√		
Cactaceae	*Opuntia ficus-indica* (L.) Miller	PAN	Per.	Ch	√		√		√	
Caryophyllaceae	*Dianthus strictus* Banks & Sol.	ME	Per.	Th				√	√	
	Spergula fallax (Lowe) E.H.Krause	ME+SA+SZ	Ann.	Th			√	√		
	Spergularia bocconei (Scheele) Aschers. & Graebn.	ME+ES	Ann.	Th			√	√		
	Spergularia diandra (Guss.) Heldr. & Sart.	ME+IT+SA	Ann.	Th			√	√		√
	Spergularia marina (L.) Griseb.	PT	Ann.	Th			√	√		
Asteraceae	*Artemisia sieberi* Besser	IT	Per.	Ch				√	√	
	Centaurea sinaica DC.	SA	Ann.	Th	√	√	√	√	√	√
	Centaurothamnus maximus (Forssk.)	SA	Per.	Ch				√		
	Erigeron canadensis (L.) Cronquist.	AM	Ann.	Th	√		√	√	√	
	Echinops spinosus L.	IT	Per.	He	√	√	√	√	√	√
	Euryops arabicus Steud. ex Jaub. & Spach.	SA+SU+SI	Per.	Th	√		√	√		
	Felicia abyssinica Schi.-Bip.	SA	Per.	Ch					√	
	Flaveria trinervia (Spreng.) Mohr	SU+ZA	Ann.	Th						√
	Lactuca serriola L.	ME+IT+ES+SZ	Bi.	Th	√		√	√		√
	Launaea intybacea (Jacq.) Beauverd	PAN	Ann.	Th			√			

	Osteospermum vaillantii (Decne.) Norl.	SA+SU	Per.	Ch					√	
	Pluchea dioscoridis (L.) DC.	SA+SU+ZA+SI	Per.	Ph	√		√	√		√
	Psiadia punctulata (DC.) Vatke	SU+ZA+SA	Per.	Ch	√	√	√	√	√	
	Pulicaria arabica (L.) Cass.	ME+IT	Ann.	Th			√	√	√	√
	Pulicaria undulata (L.) C.A. May	SA	Per.	Ch	√	√	√	√	√	√
	Picris babylonica Hand-Mazz.	SA	Ann.	Th			√	√	√	
	Scorzonera musilii Vel.	ME	Ann.	He	√					
	Senecio glaucus L.	SA+IT	Ann.	Th	√		√	√	√	√
	Senecio vulgaris L.	IT+ES+ME	Ann.	Th	√		√	√	√	√
	Silybum marianum (L.) Gaert.	ME+IT	Bi.	Th			√			
	Sonchus oleraceus L.	ES+ME+IT	Ann.	Th	√		√	√	√	√
	Verbesina encelioides (Cav.) Benth. & Hook.	PAN	Ann.	Th	√	√	√	√	√	√
	Xanthium strumarium L.	PT	Ann.	Th	√					√
Convolvulaceae	*Convolvulus arvensis* L.	COSM	Per.	Ge	√					
Cucurbitaceae	*Citrullus colocynthis* (L.) Schrader	SA	Per.	He	√				√	√
Cupressaceae	*Juniperus procera* Hochst ex. Endl.	ME	Per.	He		√	√	√	√	
Cyperaceae	*Cyperus laevigatus* L.	ME+SA+IT	Per.	He			√	√		
	Cyperus rotundus L.	PAN	Per.	Ge	√					√
	Eleocharis geniculata (L.) Roem. Schult.	PAN	Ann.	He			√	√		√
Euphorbiaceae	*Euphorbia prostrata* Aiton	COSM	Ann.	Th	√		√			√
	Ricinus communis L.	STR	Per.	Ph			√	√		√
Fabaceae	*Acacia ehrenbergiana* Hayne	SA+SZ	Per.	Ph	√	√	√	√	√	√
	Acacia gerrardii Benth.	SA+SI	Per.	Ph	√					√
	Astragalus atropilosus (Hochst.) Bunge	SZ	Per.	He		√			√	
	Indigofera spinosa Forssk.	SA+SZ+SI	Per.	Ch	√					√
	Prosopis juliflora (SW.) DC.	SA+SZ	Per.	Ph						√
	Tephrosia purpurea ssp.	SU+AF	Per.	Ch		√		√	√	√
Frankeniaceae	*Frankenia pulverulenta* L.	ES+ME+IT	Ann.	Th			√			
Juncaceae	*Juncus bufonius* L.	COSM	Ann.	Th			√	√		
Lamiaceae	*Lavandula dentata* L.	ME	Per.	Ch				√	√	
	Lavandula pubescens Decne.	SA+SU	Per.	Ch	√	√	√	√	√	√
	Marrubium vulgare L.	ME+IT	Per.	Ch			√	√		
	Mentha longifolia L.	ES+ME+IT	Per.	He	√		√	√		√
	Orthosiphon thymiflorus (Roth) Steesen	ME	Per.	Th			√	√		
	Otostegia fruticosa ssp. *schimperi*	SA+SI+SZ	Per.	Th		√				√
Loranthaceae	*Plicosepalus curviflorus* (Oliv.) Tiegham	SA+SI+SZ	Per.	Ep	√					√
Lythraceae	*Lythrum hyssopifolium* L.	IT	Ann.	Th			√			
	Punica granatum L.	IT+ES	Per.	Ph			√			
Malvaceae	*Abutilon bidentatum* A. Rich.	SU	Per.	CH				√		
	Grewia tenax (Forssk.) Fiori	TR	Per.	Ph		√				
	Malva parviflora L.	ME+IT	Ann.	Th	√		√	√		√
Menispermaceae	*Cocculus pendulus* (J.R. & G. Forster) Diels	SA+SZ	Per.	Ch	√					√
Moraceae	*Ficus palmata* Forssk.	SA+SU	Per.	Ph			√			
Myrtaceae	*Eucalyptus camaldulensis* Dehn.	AUST	Per.	Ph						√
Nyctaginaceae	*Commicarpus mistus* Thulin	SZ	Ann.	Ch	√	√		√	√	√
Oleaceae	*Olea europaea* ssp. *cuspidata* (Wall. ex G. Don) Cifferi	ME	Per.	Ph		√		√	√	
Oxalidaceae	*Oxalis corniculata* L.	COSM	Ann.	He			√	√		
Papaveraceae	*Argemone ochroleuca* Sweet.	TR	Ann.	Th	√	√	√	√	√	√
Peraceae	*Clutia lanceolata* Forssk.	SA+AF	Per.	Ch			√		√	
Plantaginaceae	*Plantago amplexicaulis* Cav.	SA	Ann.	Th	√	√		√	√	
	Plantago boissieri Hausskn. & Bornm.	SA+IT	Ann.	Th			√			
	Plantago major L.	ES+ME+IT	Ann.	He	√		√	√		
	Veronica anagallis-aquatica L.	COSM	Ann.	He			√	√		
Poaceae	*Aristida mutabilis* Trin. & Rupr.	SA+IT	Ann.	Th	√	√	√	√	√	√
	Avena fatua L.	COSM	Ann.	Th		√	√	√	√	
	Bromus diandrus Roth	ME	Ann.	Th	√	√			√	√
	Cenchrus ciliaris L.	SA+SZ	Ann.	He	√	√	√	√	√	√
	Cynodon dactylon (L.) Pers.	COSM	Ann.	G	√		√	√	√	√
	Dactyloctenium aegyptium (L.) Willd.	TR	Ann.	Th	√		√		√	
	Eragrostis papposa (Roemer& Schultes) Steudel	PAN	Ann.	Th		√	√	√	√	

	Eragrostis pilosa (L.) P. Beauv.	PT	Ann.	Th		√		√		
	Foeniculum vulgare Mill.	ME+IT	Per.	He	√				√	
	Hyparrhenia hirta (L.) Stapf	ME+SA+IT	Per.	Th	√	√	√	√	√	√
	Lolium perenne L.	ME+ES+IT	Ann.	He	√		√	√	√	
	Panicum antidotale Retz.	TR	Per.	He						√
	Pennisetum setaceum (Forssk.) Chiov.	ME+PAL	Per.	He	√	√	√	√	√	√
	Poa annua L.	ME+ES+IT	Ann.	Th	√	√	√	√	√	√
	Polypogon monspeliensis (L.) Desf.	ME+SA+IT	Ann.	Th	√		√	√	√	√
	Polypogon viridis (Gouan) Breistr.	ME+IT	Ann.	He	√		√	√		
	Schismus arabicus Nees.	IT+SA	Ann.	Th	√		√	√	√	√
	Setaria verticillata (L.) P.Beauv.	PT	Ann.	Th	√		√			√
	Sporobolus festivus Hochst. ex A. Rich.	SZ	Ann.	Th		√			√	
	Stipagrostis plumosa (L.) Munro ex T. Anders.	SA+IT	Ann.	He		√			√	
	Themeda triandra Forssk.	COSM	Per.	Ge			√	√	√	
Polygalaceae	*Polygala negevensis* Danin	SA	Per.	Ch					√	
Polygonaceae	*Rumex vesicarius* L.	SA+ME	Ann.	Th	√		√		√	
Portulacaceae	*Portulaca oleracea* L.	COSM	Ann.	Th			√	√		√
Pteridaceae	*Adiantum capillus-veneris* L.	ES+ME+IT	Per.	He			√			
Primulaceae	*Anagallis arvensis* L.	ES+ME+IT	Ann.	Th				√		
	Samolus valerandi L.	PAL	Per.	He			√	√		
Resedaceae	*Caylusea hexagyna* (Forssk.) M. L. Green	SU	Ann.	Th				√	√	
	Ochradenus baccatus Del.	SA	Per.	Ph	√	√	√	√	√	√
Rhamnaceae	*Sageretia thea* (Osbeck) M.C. Johnston	SA+IT	Per.	Ph			√	√		
Sapindaceae	*Dodonaea angustifolia* L.f.	SA+SU	Per.	Ph		√		√	√	√
Scrophulariaceae	*Buddleja polystachya* Fresen.	TR	Per.	Ph				√	√	
Solanaceae	*Datura innoxia* Mill.	COSM	Ann.	Th			√	√	√	√
	Lycium shawii Roem. & Schult.	SA+SZ+IT	Per.	Ph	√	√		√	√	√
	Nicotiana glauca R.C. Graham	PAN	Per.	Nph	√	√	√	√	√	√
	Solanum incanum L.	SZ	Per.	Ch	√	√	√	√	√	√
	Solanum nigrum L.	ME+ES+IT	Per.	Th				√	√	
	Solanum surattense Burm.f.	Me	Per.	Th						√
	Withania somnifera (L.) Dun.	ME+ES+IT	Per.	Ch	√	√	√	√	√	√
Tamaricaceae	*Tamarix aphylla* (L.) Karst.	SU	Per.	Ph			√			√
Typhaceae	*Typha domingensis* (Pers.) Poir	ME+IT+PAL	Per.	He			√	√		√
Urticaceae	*Forsskaolea tenacissima* L.	SA+SZ	Per.	Ch	√	√	√	√	√	√
Verbenaceae	*Verbena officinalis* L.	COSM	Per.	Th			√	√		
	Lantana rugosa Thunb.	PT	Per.	Ch		√				
Zygophyllaceae	*Fagonia bruguieri* DC.	SA+IT	Per.	Ch	√	√	√	√	√	√
	Peganum harmala L.	ME+SA+IT+ES	Per.	Ch	√		√			√
	Tribulus macropterus Boiss.	SU	Ann.	He	√	√	√		√	√
	Tetraena simplex (L.) Beier & Thulin	SU+SA	Ann.	Th						√

WHT: Alwaht, SHFA: Ash-shafa, RDF: Ar-Ruddaf (RDF), Per.: Perrenial, Ann.: Annual, ME: Mediterranean, COSM: Cosmopolitan, SA: Saharo-Arabian, AM: American, TR.: Tropical, ES: Euro-Siberian, IT: Irano-Turanian, AU: Australian, SZ: Sudano-Zambezian, Pan: Pantropical, PAL: Palaeotropical. Life forms: Ch: Chamaephytes, He: Hemicryptophytes, Nph: phanerophytes, Th: Therophytes.

Table S2. Vegetation composition of the studied locations invaded by *Nicotiana glauca* in Taif region, western of Saudi Arabia.

No	Botanical name	WHT 1		WHT 2		SHFA 1		SHFA 2		SHFA 3		RDF	
		U	O	U	O	U	O	U	O	U	O	U	O
1	*Abutilon bidentatum* A. Rich.	-	-	-	-	-	-	-	1.29	-	-	-	-
2	*Acacia ehrenbergiana* Hayne	2.38*	-	1.80	-	-	-	-	-	-	-	1.42	1.64
3	*Acacia gerrardii* Benth.	-	-	-	-	0.71	0.56	1.46	0.97	-	-	-	-
4	*Adiantum capillus-veneris* L.	-	-	-	-	-	-	2.18	-	-	-	-	-
5	*Aerva javanica* (Burm.f.) Juss. ex Schultes	-	2.33	16.80	9.62	0.71	-	-	-	-	-	3.68	4.37
6	*Aizoon canariense* L.	8.33	5.60	-	18.78	-	-	-	-	19.36	-	15.58	8.73
7	*Argemone ochroleuca* Sweet.	-	-	-	1.04	-	0.56	-	-	-	-	-	-
8	*Aristida mutabilis* Trin. & Rupr.	6.80	2.72	8.70	4.77	2.12	1.12	-	1.93	2.26	5.48		
9	*Astragalus atropilosus* (Hochst.) Bunge	-	-	-	-	-	-	-	-	1.94	-	-	
10	*Atriplex leucoclada* Boiss.	-	-	-	-	-	-	-	-	-	-	-	4.37
11	*Avena fatua* L.	-	-	-	-	1.42	-	-	-	-	-	-	-
12	*Blepharis attenuata* Napper.	-	-	-	4.70	-	-	-	-	-	-	-	-
13	*Brassica tournefortii* Guan.	-	-	-	-	-	1.69	-	-	-	-	-	-
14	*Bromus sericeus*	-	3.50	-	-	-	-	-	-	-	-	-	-
15	*Calotropis procera* (Ait.) Ait.f.	-	-	-	-	-	-	-	-	-	-	-	1.09
16	*Caylusea hexagyna* (Forssk.) M. L. Green	-	-	-	1.04	2.83	2.36	3.28	1.45	-	-	-	-
17	*Cenchrus ciliaris* L.	2.38	6.61	7.20	4.96	2.12	3.37	-	2.58	1.45	5.25	5.19	5.73
18	*Centaurea pseudosinaica* Czerep.	-	-	-	-	3.89	1.20	-	1.93	2.90	-	-	-
19	*Cheilanthes vellea* (Aiton) Domin	-	-	-	-	-	-	-	2.42	-	-	-	-
20	*Chenopodium carinatum* R.Br.	3.57	4.08	-	-	1.42	2.25	1.09	3.48	-	-	-	-
21	*Chenopodium glaucum* L.	-	-	-	-	1.42	7.31	0.00	1.93	-	-	-	-
22	*Chenopodium murale* L.	-	2.33	-	-	2.83	5.34	9.18	3.63	10.65	-	-	1.91
23	*Chenopodium vulvaria L.*	8.93	-	-	-	-	-	-	-	-	-	-	1.09
24	*Citrullus colocynthis* (L.) Schrader	-	-	-	-	-	-	-	-	-	-	-	1.09
25	*Cocculus pendulus* (J.R. & G. Forster) Diels	-	-	-	-	-	-	-	-	-	-	2.12	1.09
26	*Commicarpus mistus* Thulin	5.95	1.75	7.20	1.04	-	-	2.73	-	-	-	-	-
27	*Convolvulus arvensis* L.	4.08	3.79	-	-	-	2.81	-	-	-	-	-	-
28	*Erigeron bonariensis* L.	-	3.50	-	-	7.43	3.00	-	3.87	3.55	-	-	-
29	*Cynodon dactylon* (L.) Pers.	2.86	7.00	-	-	26.74	17.67	7.04	8.49	18.72	19.99	9.71	7.49
30	*Cyperus laevigatus* L.	-	-	-	-	-	-	2.18	-	-	-	-	-
31	*Dactyloctenium aegyptium* (L.) Willd.	1.19	-	9.00	6.68	-	-	-	-	-	-	-	-
32	*Datura innoxia* Mill.	-	-	-	-	2.12	0.84	-	1.93	-	-	2.83	3.27
33	*Dianthus strictus* Banks & Sol.	-	-	-	-	-	-	-	-	-	5.48	-	-
34	*Dodonaea angustifolia* L.f.	-	-	-	-	-	-	-	0.97	-	1.37	-	-
35	*Echinops spinosus* L.	-	-	-	-	1.27	1.59	2.73	1.69	-	7.76	-	-
36	*Eragrostis papposa* (Roemer& Schultes) Steudel	5.95	1.17	9.90	3.13	2.12	2.25	3.00	4.03	5.93	10.50	-	-
37	*Euphorbia prostrata* Aiton	2.38	-	-	-	-	-	-	-	-	-	-	-
38	*Euryops arabicus* Steud. ex Jaub. & Spach.	-	-	-	-	-	-	6.28	2.42	-	-	-	-
39	*Fagonia bruguieri* DC.	1.19	-	-	5.22	-	-	-	-	0.97	4.11	8.26	6.00
40	*Farsetia longisiliqua* Decne.	1.79	3.50	-	-	-	-	-	-	1.94	-	-	-
41	*Felicia abyssinica* Schi.-Bip.	-	-	-	-	-	-	-	-	-	2.05	-	-
42	*Flaveria trinervia* (Spreng.) Mohr	-	-	-	-	-	-	-	-	-	-	2.83	2.18
43	*Foeniculum vulgare* Mill.	-	-	-	-	-	0.56	-	-	-	-	-	-
44	*Forsskaolea tenacissima* L.	1.19	-	5.40	10.85	4.25	-	-	-	-	-	-	3.27
45	*Frankenia pulverulenta* L.	-	-	-	-	-	-	-	-	-	-	7.08	2.73
46	*Halothamnus bottae* Jaub. & Spach.	-	-	4.05	3.13	-	-	-	-	-	-	-	2.18
47	*Heliotropium curassavicum* L.	-	-	-	-	1.42	-	-	-	-	-	10.38	7.10
48	*Hyparrhenia hirta* (L.) Stapf	2.21	4.67	3.60	3.13	1.84	2.53	3.71	2.71	3.98	7.30	-	-
49	*Hypoestes forsskaolii* (Vahl) R.Br.	-	-	-	-	1.42	-	-	3.14	-	-	-	-
50	*Juncus bufonius* L.	-	-	-	-	-	3.65	-	2.58	-	-	-	-
51	*Lactuca serriola* L.	-	-	-	-	3.36	2.36	4.37	1.93	3.87	-	-	-
52	*Lavandula pubescens* Decne.	-	-	1.80	9.39	1.42	1.69	5.19	3.87	-	-	-	-

53	*Lycium shawii* Roem. & Schult.	-	-	-	-	-	-	-	-	-	-	-	1.09
54	*Malva parviflora* L.	-	-	-	-	4.96	1.69	6.01	3.87	-	-	-	-
55	*Marrubium vulgare* L.	-	-	-	-	1.42	0.94	2.18	1.93	-	-	-	-
56	*Mentha longifolia* L.	-	-	-	-	-	1.12	1.09	-	-	-	-	-
57	*Ochradenus baccatus* Del.	-	-	-	1.04	-	-	-	-	-	-	1.42	-
58	*Osteospermum vaillantii* (Decne.) Norl.	-	-	-	-	-	-	-	-	4.36	4.26	-	-
59	*Opuntia ficus-indica* (L.) Miller	-	-	-	-	-	-	-	-	-	1.37	-	-
60	*Panicum antidotale* Retz.	-	-	-	-	-	-	-	-	-	-	-	1.09
61	*Peganum harmala* L.	-	-	-	-	-	-	-	-	-	-	4.72	5.64
62	*Pennisetum setaceum* (Forssk.) Chiov.	-	-	-	-	-	-	2.18	1.93	-	2.74	2.83	1.82
63	*Plantago amplexicaulis* Cav.	-	-	-	-	-	-	3.28	-	-	-	-	-
64	*Plantago major* L.	-	-	-	-	-	-	7.10	3.87	-	-	-	-
65	*Poa annua L.*	-	-	-	-	-	-	7.65	4.83	-	-	-	-
66	*Polygala negevensis* Danin.	-	-	-	-	-	-	-	-	0.97	1.37	-	-
67	*Polypogon monspeliensis* (L.) Desf.	-	-	-	-	-	0.84	1.09	1.93	-	-	-	1.09
68	*Polypogon viridis* (Gouan) Breistr.	3.57	2.33	-	-	1.42	2.25	-	-	-	-	-	-
69	*Portulaca oleracea* L.	-	-	-	1.04	-	-	-	-	-	3.19	-	2.18
70	*Psiadia punctulata* (DC.) Vatke	-	1.17	1.80	-	-	-	-	1.45	-	-	-	-
71	*Pulicaria arabica (L.) Cass.*	-	-	-	-	2.36	5.27	3.28	4.35	-	-	-	-
72	*Pulicaria undulata (L.) C.A. May*	2.38	2.33	3.60	4.17	3.54	3.02	6.24	5.16	-	-	2.83	4.37
73	*Rumex vesicarius* L.	-	-	-	-	-	-	-	-	-	2.05	-	-
74	*Salsola kali* L.	7.38	4.43	4.50	1.04	-	2.53	-	3.38	6.99	9.58	-	1.09
75	*Samolus valerandi* L.	-	-	-	-	-	1.12	-	1.93	-	-	-	-
76	*Schismus arabicus* Nees.	2.38	-	-	-	-	1.97	-	-	-	-	-	-
77	*Scorzonera musilii* Vel.	-	3.50	-	-	-	-	-	-	-	-	-	-
78	*Senecio sumarae* Deflers.	-	-	-	-	-	1.12	-	-	-	-	-	-
79	*Setaria verticillata* (L.) P.Beauv.	-	-	-	-	-	-	-	-	-	-	-	5.19
80	*Solanum incanum* L.	1.79	4.67	-	4.17	-	-	-	-	1.94	-	2.83	1.64
81	*Solenostemma argel* (Del.) Hayne	-	1.17	2.52	1.04	-	-	-	-	-	-	-	-
82	*Sonchus oleraceus* L.	-	1.17	-	-	0.71	-	-	-	-	-	-	-
83	*Stipagrostis hirtigluma* (Trin. & Rupr.) De Winter	2.38	3.50	1.80	-	-	-	-	-	-	2.74	-	-
84	*Tamarix aphylla* (L.) Karst.	-	-	-	-	-	0.56	-	-	-	-	2.12	2.91
85	*Tephrosia purpurea* ssp.	-	-	-	-	-	-	-	-	-	1.37	-	-
86	*Tetraena simplex* (L.) Beier & Thulin	-	-	-	-	-	-	-	-	-	-	-	3.27
87	*Themeda triandra* Forssk.	-	-	-	-	-	0.56	-	1.93	-	-	-	-
88	*Tribulus macropterus* Boiss.	6.19	12.11	-	-	-	1.12	3.28	2.90	-	-	4.25	1.09
89	*Typha domingensis* (Pers.) Poir	-	-	-	-	-	1.12	-	1.29	-	-	2.83	-
90	*Verbena officinalis* L.	-	-	-	-	4.96	1.12	-	-	-	-	-	-
91	*Verbesina encelioides* (Cav.) Benth. & Hook.	7.54	4.43	-	-	0.71	-	-	-	-	-	-	2.18
92	*Veronica anagallis-aquatica* L.	-	-	-	-	2.12	-	-	-	-	-	-	-
93	*Withania somnifera* (L.) Dun.	1.19	3.50	-	-	2.12	4.12	-	-	1.94	2.05	-	-
94	*Xanthium strumarium* L.	-	-	-	-	1.42	0.56	-	-	-	-	-	-

* values are mean of the relative density, WHT: Alwaht, SHFA: Ash-shafa, RDF: Ar-Ruddaf (RDF).

Table S3. Coordinates and altitudes of different locations invaded by *Nicotiana glauca* in Taif region, western of Saudi Arabia.

**Site**	**Locations**	**Lat/long**	**Elevation (m.a.s.l.)**
Alwaht	WHT1	21°10'05" N – 40°21'24" E	1819
	WHT2	21°09'39" N – 40°21'38" E	1865
Ash-shafa	SHFA1	21°04'14" N – 40°21'30" E	2059
	SHFA2	21°04'33" N – 40°21'44" E	2078
	SHFA3	21°04'54" N – 40°22'10" E	2046
Ar Ruddaf	RDF	21°13'22" N – 40°26'18" E	1674

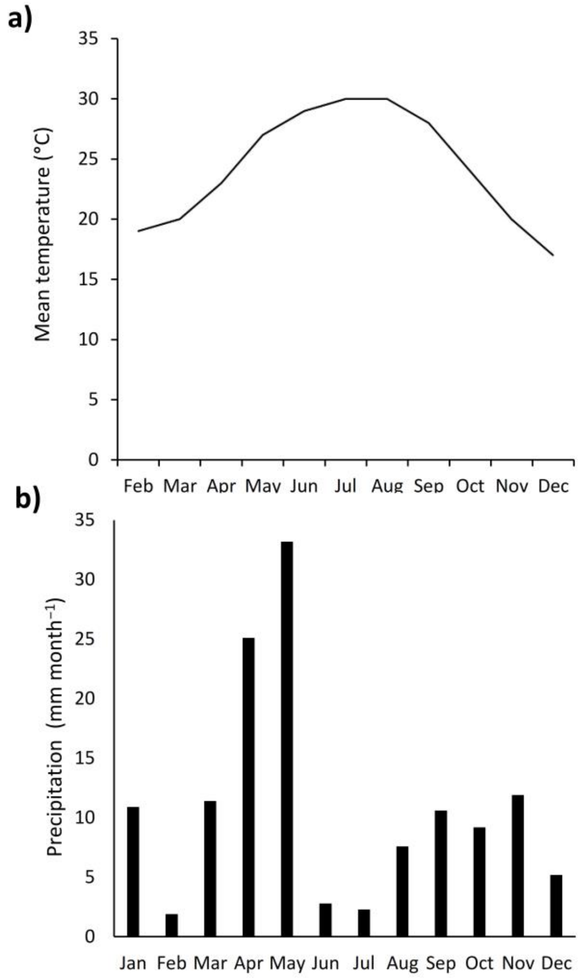

Figure S1. The data are long-term averages, based on weather reports during 2005–2015, recorded at the Taif meteorological station. (**a**) mean temperature (°C) and (**b**) precipitation (mm month^{-1}).
